# Reply: Height, body mass index, and prostate cancer – a follow-up of 950 000 Norwegian men

**DOI:** 10.1038/sj.bjc.6601801

**Published:** 2004-04-06

**Authors:** A Engeland, S Tretli, T Bjørge

**Affiliations:** 1 Division of Epidemiology, Norwegian Institute of Public Health, PO Box 4404, Nydalen, N-0403 Oslo, Norway

**Sir**,

We agree with Dr Batty that information on other variables could have strengthened our study of height in relation to the incidence of prostate cancer. Body height is previously found correlated to socioeconomic status. However, we do not think that the suggested variable on socioeconomic position could explain the observed relation between height and prostate cancer incidence in our cohort. An analysis on Norwegian birth cohorts (Harvei and Kravdal, 1997) showed that men with occupations connected to high education had less than 30% higher risk of prostate cancer than men in occupations connected to low education. In the Finnish publication (Pukkala and Weiderpass, 2002) referenced by Dr Batty, the highest social class had 40–50% higher incidence of prostate cancer than the lowest social class. According to Bross (1967), a much larger effect on potential confounding variables is needed to explain as large effects as observed in our study (men with tall stature had 70% as large risk of prostate cancer as the lowest men).

Dr Batty claims that one Norwegian cohort study (Lund Nilsen and Vatten, 1999) showed that the magnitude of the association between height and prostate cancer was attenuated following control for social factors. This is not correct. Lund Nilsen and Vatten (1999) wrote that the associations between the anthropometrical variables (height and BMI) and prostate cancer risk were not confounded by the factors included in the multivariate analysis (smoking status, physical activity, educational attainment and marital status). Neither the other cohort study (Leon *et al*, 1995), Dr Batty refers to, shows that adjusting for socioeconomic status attenuates the association between height and prostate cancer as argued.
